# Comparison of Tool Wear, Surface Roughness, Cutting Forces, Tool Tip Temperature, and Chip Shape during Sustainable Turning of Bearing Steel

**DOI:** 10.3390/ma16124408

**Published:** 2023-06-15

**Authors:** Havva Demirpolat, Rüstem Binali, Abhishek D. Patange, Sujit S. Pardeshi, Sakthivel Gnanasekaran

**Affiliations:** 1 Department of Mechanical Engineering, Faculty of Technology, Selcuk University, Konya 42130, Turkey; hdemirpolat@selcuk.edu.tr; 2 Department of Mechanical Engineering, COEP Technological University Pune, Pune 411005, Maharashtra, India; 3 Centre of Automation, School of Mechanical Engineering, Vellore Institute of Technology, VIT, Chennai 600127, Tamil Nadu, India

**Keywords:** AISI 52100, turning, MQL, dry machining

## Abstract

In this study, a comparison of measured cutting parameters is discussed while machining AISI 52100 low-alloy hardened steel under two different sustainable cutting environments, those in which a dry and minimum quantity lubrication (MQL) medium are used. A two-level full factorial design method has been utilized to specify the effect of different experimental inputs on the turning trials. Experiments were carried out to investigate the effects of three basic defining parameters of turning operation which are namely cutting speed, cutting depth, feed rate effects and also the effects of the cutting environment. The trials were repeated for the combination of different cutting input parameters. The scanning electron microscopy imaging method was used to characterize the tool wear phenomenon. The macro-morphology of chips was analyzed to define the influence of cutting conditions. The optimum cutting condition for high-strength AISI 52100 bearing steel was obtained using the MQL medium. The results were evaluated with graphical representations and they indicated the superiority of the pulverized oil particles on tribological performance of the cutting process with application of the MQL system.

## 1. Introduction

For decades, low-alloy hardened steels for their superior properties have been widely used in manufacturing. The increasing use of these materials requires high-performance machining processes, which constantly present new challenges for machining processes. Much research has been conducted to enhance the machining outputs of low-alloy steel [1,2,3,4]. The machining of low-alloy hardened steels (>45 HRC) is referred as to hard machining especially when used in the single-point turning of bearings [5]. AISI 52100-grade, high-chromium, carbon-containing low-alloy steel is called bearing steel which is characterized by dimensional stability, abrasion resistance, and high strength [5,6]. The hard turning of bearing steel provides an economical solution with high material removal rates instead of grinding. However, AISI 52100 bearing steel is a difficult-to-machine material owing to its high strength. Additionally, a high temperature between the tool, workpiece and chip causes a poor surface finish and tool life. Cutting fluid is an effective solution, keeping the temperature that results during machining under control so that surface roughness, cutting force and tool wear decrease. However, flooding cooling methods have dangerous effects on the environment and can be harmful to human health. They also increase production costs and may require extra operations such as post-processing cleaning [7]. Moreover, green cutting technology is a new concept that can respond well to strict environmental regulations [8]. By implementing green cutting technology, industries can reduce their carbon footprint and contribute to a cleaner and healthier environment. Additionally, the adoption of green cutting technology can improve the quality of life for workers in the industry by reducing their exposure to harmful pollutants and improving workplace safety. Overall, green cutting technology is a promising solution for industries looking to meet environmental regulations and promote sustainable practices. Dry machining is an alternative machining process that has many advantages economically and environmentally [9]. Dry machining can also improve efficiency and productivity by reducing machine downtime associated with fluid changes and cleaning. Cutting fluids can often cause issues with chip disposal and can increase the frequency of machine maintenance, while dry machining produces dry and easy-to-dispose-of chips. MQL is another lubricating method in which a coolant is sent to the cutting zone effectively [10]. However, with MQL, the volume of coolant is significantly reduced, typically to the range of 30–100 mL/h, and is applied directly to the cutting edge with precision nozzles. This reduces the amount of lubricant needed, resulting in lower costs, less waste, and reduced environmental impact. MQL also improves the overall machining process by reducing chip welding, improving surface finishes, and extending tool life. In the literature, research has been performed on the machining of AISI 52100 under different environmental conditions [11,12,13,14]. An experimental study was performed by Biček et al. [15] to determine the cutting environment effects on machining performance. Cryogenic cooling methods demonstrated superior cutting performance when compared to dry and flooding methods. Rajarajan et al. [16] performed a comparative study on bearing steel characteristics in machining. Machining experiments were optimized using the Taguchi technique. The authors concluded that cutting temperature is an important parameter affecting surface integrity. Mia et al. [17] stated that the depth of a cut is the main parameter that determines the characterization in hard machining in MQL environments. The metallurgical structure of a hard-machined surface is another important machining aspect. Hard turning can introduce on the machined surface a transformed nanocrystalline layer less than one micron in depth from the surface called a white layer [18]. The appearance of a white layer is a phenomenon caused by microstructural alteration in micro-scale tool and workpieces [19]. A microstructurally altered layer may increase in area with increased tool flank wear. Therefore, tool wear plays an important role in the hard turning of AISI 52100, which prevents residual stress and surface integrity. Shibab et al. [20] investigated the different cutting input parameter effects on surface quality in turning bearing steel in dry machining. The results showed that the important cutting parameter is feed rate, while the effects of cutting speed and the depth of cut are less. Diniz et al. [21] evaluated the tool wear mechanism in the hard turning of bearing steel under dry and flood cooling conditions. Cooling conditions with the minimum volume of oil (MVO) presented better surface roughness properties than flood and dry environments did. Low-friction DLC coatings and mixed ceramic inserts were evaluated for the dry machining of AISI 52100 by Kumar et al. [22]. The results showed that coated tools reduce surface roughness and inhibit the progression of tool wear. Bhandarkar et al. [23] studied the performance of coated tools in machining bearing steel. Experiments were optimized via Taguchi–Grey relational analysis. It was found that the feed rate was the dominant parameter among the cutting parameters. Bouacha et al. [24] analyzed surface roughness and cutting force in the hard turning of AISI 52100 using the CBN tool. Rana et al. [25] evaluated the surface integrity of AISI 52100 bearing steel in machining. Cutting parameters were evaluated under the MQL cooling condition using a tungsten carbide tool. Optimum cutting parameters were determined at a cutting speed of 150 m/min, feed rate of 0.1 mm/rev, depth of cut of 0.3 mm, and flow rate of 100 mL/h. Sankar and Umamaheswar Rao [26] analyzed the effect of the turning process on bearing steel by using the Taguchi method. Depth of cut was determined as the main parameter in machining, followed by the cutting speed and feed rate for cutting. Çetindağ et al. [27] experimentally investigated the surface aspects in machining AISI 52100 under a hybrid cooling condition. It was stated that considerable improvements were obtained by the authors employing a MQL+ indirect cryogenic (CO_2_ + LN_2_) coolant. Serra et al. [28] developed a model to observe the relationship between cutting parameters and the performance of bearing steel. The results confirmed the experimental parameters. Rana et al. [29] evaluated the surface roughness in machining under the MQL condition. The experimental plan was created with the full factorial design method. Temperature and surface roughness increased with the increase in the nozzle distance and nozzle elevation angle of MQL. Sing and Rao [30] studied the effect of thermal and physical conditions of cutting on the surface quality in the hard turning of bearing steel based on tool geometry. The first-order prediction model was found suitable and the main factors affecting the surface quality were feed rate, and tool geometry. Jouini et al. [31] proposed an alternative finishing method, precision hard turning (PHT), instead of grinding AISI 52100 (60–62 HRC). The authors obtained a better surface integrity with increasing rolling contact fatigue (RCF) using the PHT method under dry conditions using CBN. A uniform white layer results in decreasing dislocations in the transition zone. An experimental study was performed by Mane and Kumar [32] to improve the machining performance of AISI 52100 using multilayer coated carbide tools in the MQL condition. The response surface methodology enhanced the 95% confidence level. In this study, where an analysis of variance (ANOVA) was employed to specify the optimum cutting parameters, feed rate was the main parameter. Özel and Karpat [33] used a neural network model to predict the machining outputs of AISI 52100 in the turning process. In this study, the authors obtained better surface properties by decreasing the feed rate.

In the present study, experiments were conducted to investigate the effects of three basic defining parameters of turning operation which are namely cutting speed, cutting depth, feed rate effects including the effects of the cutting environment. The novelty of this experimental study on turning AISI 52100 as a workpiece material in a MQL environment is that previous studies on MQL have mostly focused on aluminum alloys and other non-ferrous metals. AISI 52100 is a high-carbon, chromium-bearing alloy steel commonly used in the manufacturing of ball bearings and other machine elements. The use of MQL for machining AISI 52100 has the potential to improve surface quality, reduce tool wear, decrease machining time, and lower energy consumption. Therefore, this study can offer insights into how MQL can be extended to more challenging workpiece materials and expand its practical applications. In addition, the effect of cutting parameters of the two sustainable machining methods, dry and MQL-assisted turning, has not been previously determined for AISI 52100-grade bearing steel. Experiments were designed by employing a full factorial design method. This experimental study presents the optimum values of cutting parameters affecting surface roughness, tool wear and temperature to professionals and researchers.

## 2. Materials and Methods

AISI 52100-grade low-alloy hardened bearing steel was investigated as a workpiece in this experimental study. The chemical composition of the workpiece is presented in Table 1. The measurements of the round billet were a 30 mm diameter and 100 mm length. In the experiments, the full factorial design principle was used. Parameters used in the experiment, cutting insert, cutting speeds, and feed rates, were determined using a full factorial method across the cutting environments on different levels. This method involves testing all possible combinations of factors to specify the optimal settings for the process with minimum time and resource consumption. The purpose of using a full factorial design is to ensure that all possible interactions between the parameters are considered, which can provide more accurate and reliable results compared to those of other experimental designs in a shorter time. The full factorial design method also warrants a statistical analysis of the results, which can provide insight into the significance of effects and interactions [34]. This can help in optimizing the cutting parameters for a specific cutting environment, leading to improved machining performance and reduced costs. A total of 16 experiments were performed on the workpiece using a simple systematic design that allows an estimation of the main effects and interactions. The first step in designing the experiment was to identify the cutting factors that may affect the response. Each factor has at least two levels, and the levels were chosen based on what is relevant and reasonable for the specifics in this study. Once the factors and levels were determined, an experiment plan was created that included all possible combinations of the levels for each factor. The next step was to assign test points to each combination of factor levels. In this way, the experimental results were evaluated while testing multiple factors simultaneously. Additionally, the main effects and interactions were specified by using the full factorial design method in this study.

### 2.1. Materials of Tool and Workpiece

The cutting tool and the tool holder were selected in line with the practical applications and recommendations of the manufacturers. The TiC-coated CCMT 09T308-304-series cutting tool was used in accordance with ISO 3685 [35]. Cutting tool information is given in Table 2. Cutting tools were renewed after every trial period.

### 2.2. Machining Experiments

**Figure 1:**
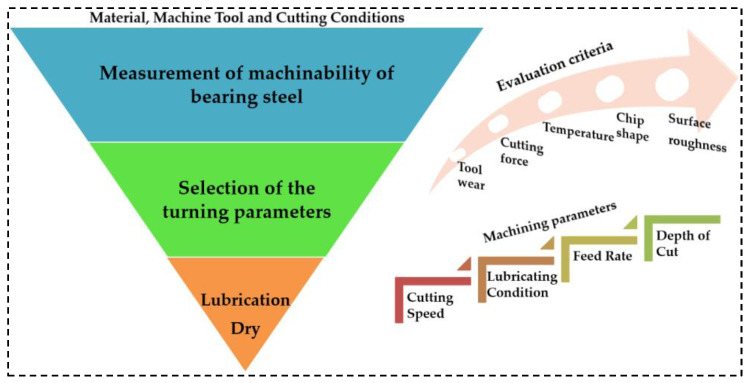
Experimental approach and utilized machining conditions during turning.

Machining trials were conducted under dry and MQL-assisted cutting environmental conditions. The experimental approach and a general overview of the study is summarized in Figure 1. The comprehensive cutting input parameters are specified in Table 3.

### 2.3. Description of Machining Conditions

The turning experiment was carried out for a cutting depth of 0.2–0.6 mm, feed rates of 0.1–0.3 mm/rev and cutting speeds of 30–90 m/min under dry and MQL environment conditions. The MQL device was utilized with a 50 mL/h flow rate condition. The MQL device was installed at a 20 mm distance to better the surface quality by controlling temperature. Mineral oil-based cutting fluid was sprayed from a nozzle with a diameter of 2 mm.

### 2.4. Measurement of Response Parameters

Surface roughness (Ra) values were measured by using a portable perthometer measuring machine (Brand: Mahr Co., Ltd., Göttingen, Germany). This instrument can detect deviations of up to 150 μm. To determine the surface roughness values of the workpiece, it was measured, revealing a 0.8 mm cutting length and 5.6 mm tracing length. The roughness value was determined by subtracting the highest and lowest values from the measurements and taking the average of the three values. In addition, cutting forces were measured using a Kistler 9275 dynamometer and signals were recorded on a computer. Temperature and tool wear monitoring can present remarkable information about cutting conditions and its environmental effects. Tool tip temperature was measured using the InGaAs (TeLC, Bad Homburg, Germany) radiation method with a sensor which was placed at a certain distance. Thermometer signals obtained from the sensor transferred them to the PC and then were evaluated. It was possible to take high-precision measurements with an accuracy rate of 0.1% by calculating current and voltage instantly during the cutting process. The progression of flank wear was measured using an optical microscope. SEM visualization was used to characterize the tool wear mechanism. The macro-morphology of chips was investigated to define the influence of the cutting conditions.

## 3. Results

Due to the complex nature of machinability studies, examining the effects of some variables on the process is necessary to evaluate the analysis meaningfully. For this reason, the findings obtained from the experimental study will be evaluated in terms of tool wear, surface roughness, cutting force, tool tip temperature, and chip shape in this section using graphic and visual materials.

Tool wear is evaluated according to VBmax as it is the limit value. Surface roughness, cutting force, and temperature values are evaluated considering the average values.

### 3.1. Tool Wear

**Figure 2:**
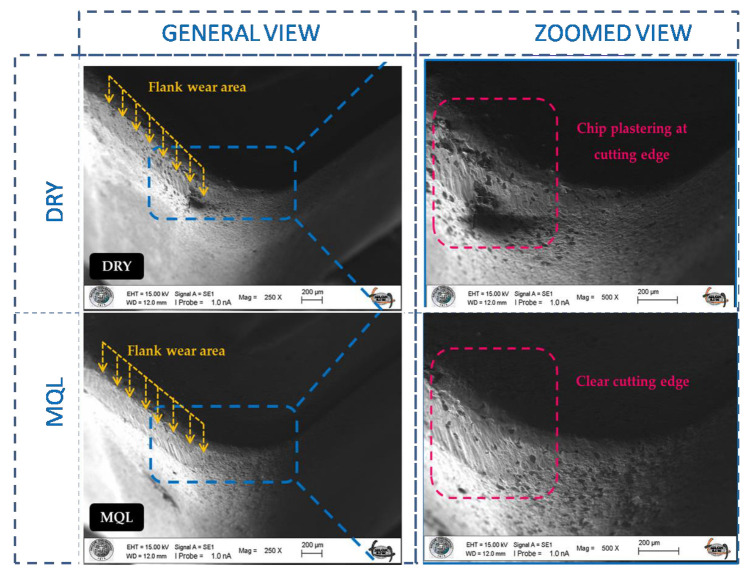
Tool wear conditions under MQL and dry medium environments during turning of bearing steel (0.3 mm/rev, 0.6 mm, 90 m/min).

Tool wear is one of the most important quality characteristics in machining operations, as it has a decisive influence on cutting edge geometry, machine surface quality, workpiece dimensions, and cutting mechanisms [36]. The evaluation of tool life is mainly based on predicted surface damage in the hard turning of hardened low-alloy steel [37]. Flank wear is the dominant wear type in the turning of AISI 52100. Flank wear is generally accepted as the determiner of tool life as tool wear progresses on tool faces [38]. As flank wear progresses on the tool face, the cutting tool loses its cutting edge and ability. This condition causes an increase in cutting force and power consumption resulting in chatter vibration and the worst surface integrity. Figure 2 shows significant flank wear formed on flank faces and little rake faces. The most considerable flank wear bandwidth is detected under dry cutting conditions based on higher temperature generation at the cutting zone. Lubrication reduces the effects of wear at the interface between the tool and workpiece and reduces friction. Additionally, good penetration of oil droplets in compressed air improves cutting well. Figure 2 illustrates chip plastering at the cutting edge under dry cutting conditions. Higher temperatures in the cutting zone cause adhesion and abrasion wear mechanisms. Abrasive, adhesive, and diffusive wear formations are the main factors determining the wear texture [39]. This case also promotes diffusion so that crater wear may be formed under dry cutting conditions.

**Figure 3:**
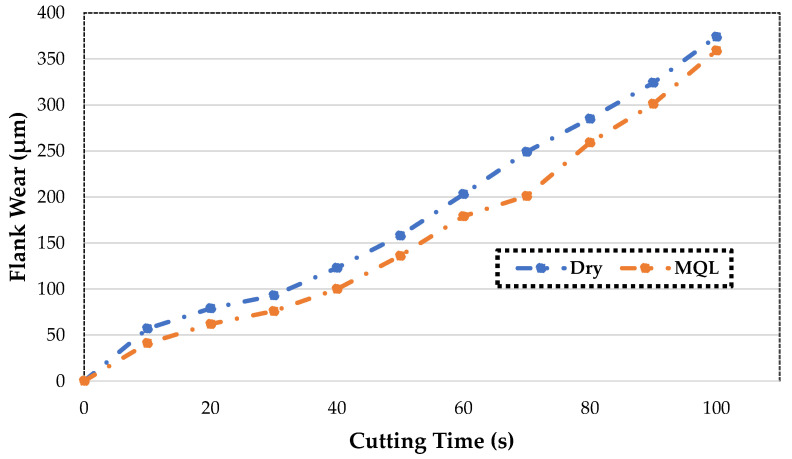
Flank wear against time for different cutting conditions.

As seen in Figure 3, the critical value is exceeded in both environments after a specific cutting time. It should be noted that the values in this chart refer to operating conditions of 0.3 mm/rev, 0.6 mm, and 90 m/min. As a result of the experiments, MQL can be said to be more resistant to the wear tendency, although the values are close to those of the dry state. The MQL environment is also better for prolonging tool life.

**Figure 4:**
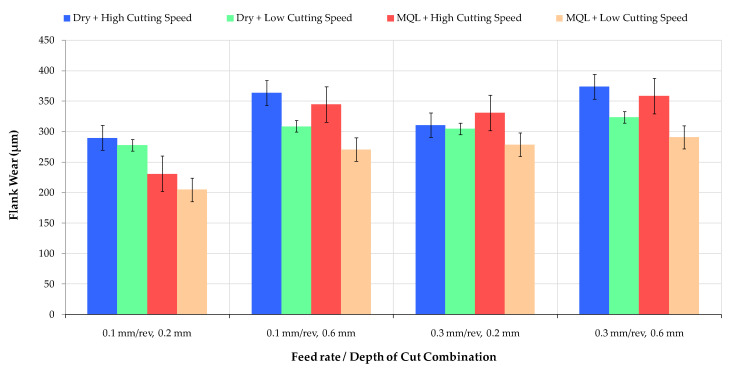
Flank wear development according to different ratios of cutting speed and lubricating media.

Figure 4 shows the overall evaluation of the effects of different cutting condition combinations on tool wear mechanisms. VBmax was used to evaluate the wear of the cutting edge. It is observed that cutting environmental conditions have a significant effect on tool flank wear progress. The worst flank wear formation is obtained under dry and high-cutting speed conditions. Flank wear indicators change linearly with an increasing cutting speed using a low feed rate and depth of cut. However, this linearity is distorted for a higher depth of cut value when using a MQL cutting medium. As seen in Figure 4, the flank wear value is lower for the low cutting speed in the dry medium than that under the MQL+ high cutting speed condition. There is an increasing relationship between cutting speed and average surface roughness.

Regarding the dry cutting condition’s flank wear value, the most significant increase of 34% was observed due to an increase in the depth of cut in high-cutting-speed machining conditions. A similar variation trend was obtained in experiments performed in an MQL medium, but the increase rate was 40%. A significant increase in flank wear was observed at a low depth of cut value (0.2 mm), depending on the feed rate increase from 0.1 mm/rev to 0.3 mm/rev in both dry and MQL conditions. This became a more apparent cut depth that significantly affected flank wear following the cutting speed in both cutting mediums. The feed rate’s effect decreased as the cut’s depth increased. Minimum flank wear was determined at a 0.1 mm/rev feed rate, 0.2 mm depth of cut, 30 m/min cutting speed, and the MQL combination. MQL-assisted machining successfully protects the tool’s flank wear and crater wear when turning AISI 52100. Thus, tool life can be increased by reducing tool wear through chip removal operations in cooling conditions.

### 3.2. Surface Roughness

**Figure 5:**
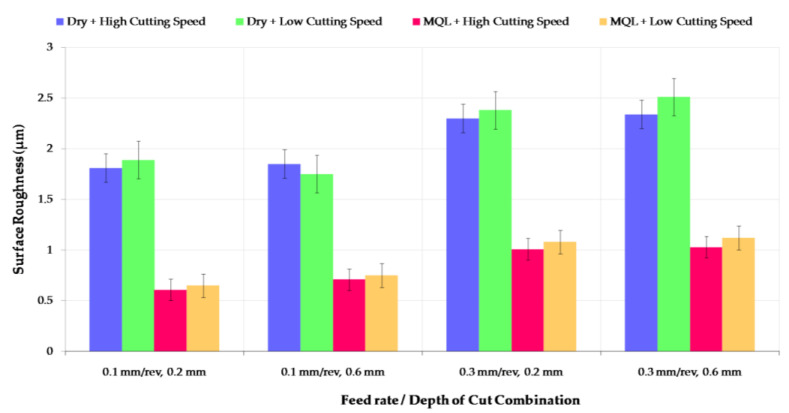
Surface roughness variations according to different ratios of cutting speed and lubricating media.

Surface roughness is a critical consideration in turning AISI 52100 because it affects the characteristics and functionality of the finished product. To achieve the desired surface roughness, it is essential to optimize these factors based on the material being turned and the required finish [40,41]. For AISI 52100, a high-quality surface finish is essential to ensure that the material meets the required standards for precision applications. Therefore, precise control over the turning process, including the choice of inserts, cutting parameters, and the environmental condition, is necessary to achieve the desired surface roughness for AISI 52100 bearing steel turning [42]. As seen in Figure 5, the Ra values vary in the range of 0.6–2.5 μm.

A poor surface finish was observed with a low cutting speed, high feed rate and high cutting depth value combination in dry machining. The lowest surface roughness values were measured under MQL conditions for all cutting parameters, especially high cutting speed. MQL cutting environment conditions provide a significant advantage compared to dry conditions in the context of surface roughness. When dry and MQL machining conditions were compared, a dramatic difference in surface roughness values was observed with a decrease from 1.89 to 0.65 at the lowest cutting parameters. A considerable amount of flank wear was seen to increase in high-cutting-speed conditions due to tool-workpiece friction, and this causes the generation of heat in the cutting region. MQL reduces tool flank wear thanks to its provision of good penetration properties, giving a better surface roughness. Depending on the cutting speed change, the surface roughness values for each cutting environment condition are very close. Surface roughness values are slightly higher for high-cutting-speed parameters. The average surface roughness values are close for cutting speed conditions, those in which MQL and dry mediums are used. However, the surface roughness difference is more evident in the dry and MQL environment. Feed rate is a significant parameter of surface roughness for both environmental conditions [43]. The surface roughness value increased by about 30% in dry conditions, but this rate is 60% on average for MQL conditions depending on the feed rate. A similar increasing trend is observed for the depth of cutting parameters, but the increase is more limited than that of the feed rate. Surface roughness is also related to chip shape; in dry conditions, ribbon-type continuous thick chips result in a poor surface for all parameters.

### 3.3. Cutting Forces

**Figure 6:**
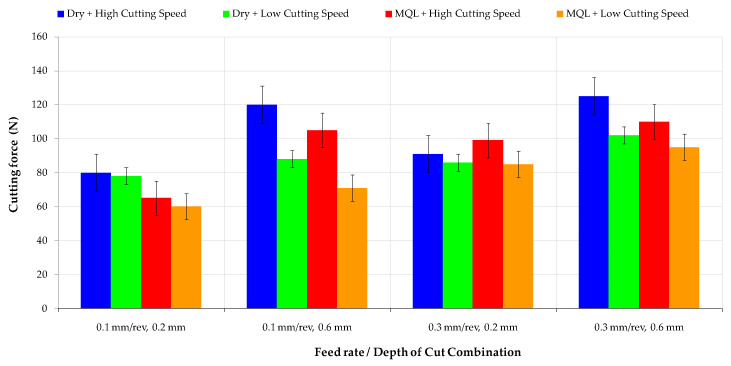
Cutting force variations according to different ratios of cutting speed and lubricating media.

AISI 52100 bearing steel has superior mechanical properties and high strength. For this reason, cutting force and power requirements are higher. Cutting force is an important parameter that determines the energy and machining process’s efficiency [44]. For sustainable manufacturing goals, the effect of long-term cutting environments on machining was investigated, these accounting for a large portion of the energy demand in manufacturing. The change in the cutting force is parallel to the tool flank wear variation in Figure 4 and Figure 6. As the cutting edge of the cutting tool becomes worn more, cutting force is required for machining. This increase in cutting force leads to increased power consumption. The maximum cutting force was found under conditions of a high cutting speed and a higher feed rate in a dry medium, as seen in Figure 6. The depth of cut is the primary parameter that increases cutting force. This observation suggests that higher feed rates and higher cutting depths result in higher resistance to cutting, requiring more force to remove material.

Furthermore, the cutting force values increased when the feed rate value was changed from minimum (0.1 mm/rev) to maximum values (0.3 mm/rev). This effect was present in both dry MQL cutting conditions, indicating that the lubrication method may not significantly affect the cutting force in this cutting combination. A generic increase trend in cutting force was observed at the higher cutting speed in the dry and MQL medium. There was a slight decrease in cutting force under low machining speeds and dry environmental conditions compared to that with MQL high-machining media in the experimental trials at high feed rates and depth of cut combinations. The cutting force values were increased by 60% on average in both cutting environments, depending on the increasing cutting depth at the same cutting speed values. Similarly, the rise in cutting force was 20% on average with the increase in feed rate from 0.1 mm/rev to 0.3 mm/rev. There was more than a 100% difference between the minimum and maximum value of cutting force. This result shows the importance of determining cutting parameters in power consumption and sustainable manufacturing goals. The optimum cutting condition for cutting force was measured during the turning of AISI 52100 bearing steel in lower cutting parameters using a MQL medium.

### 3.4. Tool Tip Temperature

**Figure 7:**
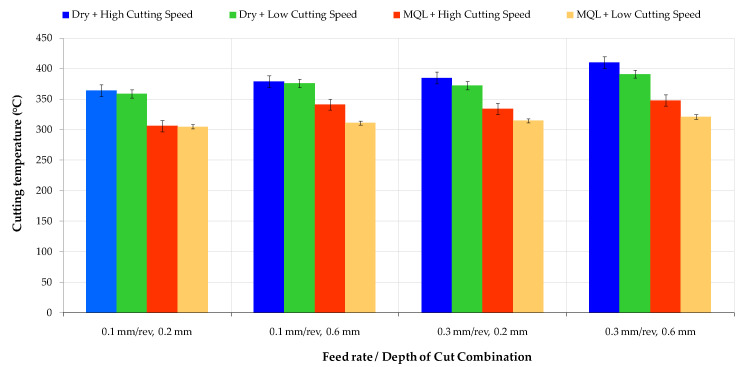
The variations om cutting temperature according to different ratios of cutting speed to lubricating media.

An increase in temperature in the cutting zone is inevitable as a result of the conversion of a large part of the mechanical energy into heat, which is caused by friction during machining [45]. The conductivity of the workpiece is another important parameter that affects the temperature variation in the cutting zone. Cutting temperatures may reach the tempering temperatures of AISI 52100, which are about 400 °C as shown in Figure 7.

Instantaneous temperature fluctuations and increases in the cutting zone are the main causes of rapid deterioration of the cutting tool. This deterioration, which progresses over time, spreads over a wider surface, affecting all cutting surfaces of the tool, and disrupts the workpiece’s surface integrity. This condition causes poor machining and material properties. Dry machining is a traditional method that does not harm the environment but shows poor performance in temperature control in cutting zones. Of course, most of the heat in the cutting region is removed from the area with chips. However, the chip that does not leave by clinging onto the surface is a secondary and very important factor that increases the temperature on the cutting surface in dry machining AISI 52100. Using cutting fluid is a solution commonly used in machining operations. In this study, the temperature reduction performance of MQL-assisted cooling was determined to be 15% on average during the AISI 52100 turning process.

### 3.5. Chip Shape

**Figure 8:**
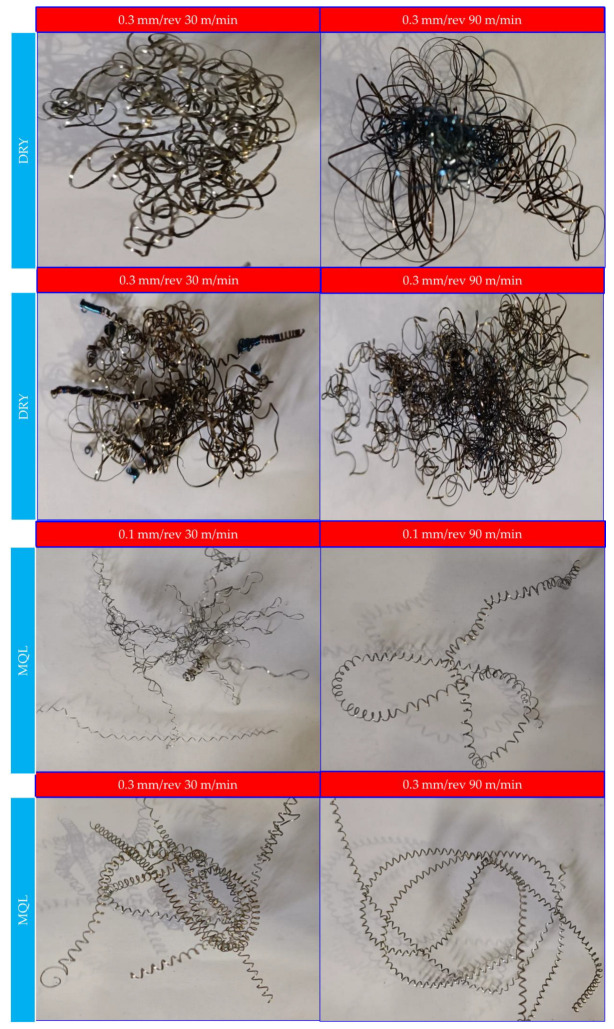
Chip shape variations according to different ratios of cutting conditions.

Chips from machining are tiny particles that break off from the workpiece material, making it possible to obtain new surfaces with desired surface quality [46]. Generally, chips formed in machining are classified by their geometric shapes, such as flowy, wavy, serrated and segmented [47]. The chip shape reflects the signs of the machining operation. Lubricating conditions and the level of cutting parameters significantly affect the shape of chips. To classify chips, many indicators can be monitored: (i) excessive heat generation, (ii) chip breakability, and (iii) tangles around the workpiece and tool. Additionally, chip morphology could be quantified to determine the effect of tool wear, cutting speed, feed rate, and depth of cut. Therefore, keeping the chip shape in a particular range can guarantee some better-quality results in machining, such as lower thermal effects, a longer tool life, better tool wear results, improved surface quality, etc. Therefore, in this study, the chips were collected after the turning operations during the machining of AISI 52100 bearing steel. Figure 8 shows the chip shapes collected under dry and MQL cutting conditions.

In this section, chip shapes are evaluated according to the ISO 3685 standard [35]. Chips of a long, tubular and snarled shape were obtained using a dry medium with low and high cutting speed parameters. It could be seen that the chip thickness slightly decreased depending on the increase in cutting speed. Tubular and washer-type chips were formed by shifting the conditions from a high to a low feed rate. However, the ribbon type was dominant at high cutting speeds and feed rate conditions; it turned into a tubular snarled type as the feed rate decreased. Irregular continuous chip tangles on the cutting edge, tool holder and machined workpiece cause poor machining aspects and surface integrity which increases the temperature in the cutting region and the vibration also. Washer-type helical chips were observed under conditions involving a MQL medium with high cutting speed and feed rate conditions. However, upon shifting the conditions from a high to low feed rate washer type helical snarled chips were observed. The chip shape change revealed the difference in plastic deformation. While the increased temperature caused chip bulk at the tooltip, most of the heat could be removed by chips.

However, while the formation of piles in the cutting zone increases the temperature, the friction of the surface increases continuously and negatively affects the surface roughness. The lower average temperatures in the MQL environment support the formation of open tubular chips at lower machining parameters. The lower surface roughness values and longer chips were obtained when AISI 52100 was machined under low cutting parameters using a MQL medium. The most enlarged chips and poor surfaces were obtained under higher cutting parameters in dry machining. Snarled chips triggered the rising temperature and poor machining in dry cutting environments. An increasing temperature due to the cutting speed and depth of cut indicates that chip forms are tighter and closed. High-carbon steel AISI 52100 gives continuous and snarled chip shapes when using a dry and MQL medium. In recent times, machine learning-based techniques have been applied for comparing various machining parameters under sustainable environments using regression and classification, and these can be considered in future work [48,49,50,51].

## 4. Conclusions

In this paper, the effects of cutting parameters and sustainable environmental conditions were investigated on the turning characteristics of AISI 52100 bearing steel. An experimental study was conducted to evaluate two sustainable cutting environments involving both MQL and dry conditions regarding tool flank wear, surface roughness, cutting force and chip shape. The MQL utilized in turning AISI 52100 resulted in significant performance improvements due to its ability to prevent an excessive temperature increase during the cutting process. This is an important finding, as high temperatures can lead to accelerated tool wear, reduced cutting speeds, and poor surface finish quality. The tool wear progressions observed for MQL-assisted turning also demonstrated better flank wear aspects when compared to those observed under dry cutting conditions. This is encouraging for industries, as it means that they can reduce their reliance on expensive coolant systems and still achieve high-quality results in their manufacturing processes.

- MQL-assisted turning prevents excessive temperature rise in turning AISI 52100 due to good penetrating properties on the cutting region. Additionally, better flank wear aspects were obtained when turning in MQL environment based on tool wear progressions. The tool flank wear differences were more pronounced at higher cutting speeds than at lower cutting speeds.
- In machining high-strength AISI 52100 bearing steel, a high-temperature region and plastic deformation led to adhesive, abrasive, and diffusive tool wear. Tool wear significantly affects the other machining outputs, cutting force, and power consumption. Tool wear, temperature, and cutting force are in close relationship. The lowest cutting force was measured at a 30 m/min cutting speed, 0.1 mm/rev feed rate, 0.2 mm depth of cut, and under the MQL condition.
- In dry conditions, a direct, more extended tool workpiece contact distance causes an increase in temperature in the cutting region. High temperatures affect the tool, chip shape, and chip removal rate. The thermal stress and shape distortions result in the worst surface roughness on the workpiece. Surface roughness is a critical consideration in turning AISI 52100 because it affects the functionality and full physical aspects of the finished product. The MQL medium presents the ideal operational conditions during AISI 52100 bearing steel turning.
- Tool tip temperatures reached 410 °C for high cutting parameters in dry machining, whereas in the MQL-assisted machining condition, a maximum tool tip temperature of 348 °C was measured at the maximum level of the cutting parameters.
- Chip shape is an essential parameter for evaluating a material’s machining properties. In the case of dry cutting, chips of a long, snarled and tangled shape build up on the tool’s cutting edge, which prevents heat removal from the cutting region. Snarled chips triggered the rising temperature, resulting in poor machining in the dry cutting environment. However, lower surface roughness values and longer chips were obtained when AISI 52100 was machined under low cutting parameters using a MQL medium since lubrication contributes to chip sliding from the cutting zone.

The results of this study indicate the potential for MQL to revolutionize the turning process, making it more efficient, cost-effective, and environmentally friendly. Future research could explore how MQL can be applied to other manufacturing processes, as well as the development of new lubricants specifically tailored for MQL conditions.

## Figures and Tables

**Table 1:** AISI 52100 bearing steel—chemical composition.

Element (wt%)	C	Mn	Si	Ni	Cr	Cu	Mo
AISI 52100	0.93–1.05	0.25–0.45	0.15–0.35	0.25	1.35–1.60	0.30	0.10

**Table 2:** Chemical composition of AISI 52100 bearing steel.

Cutting Tool	Clearance Angle	Cutting Edge Length	Tip Thickness	Corner Radius
CCMT-09T308-304	7°	9 mm	3.97 mm	0.8 mm

**Table 3:** Cutting parameters utilized in experiments.

Cutting Parameter & Its Levels	Cutting Speed (m/min)	Feed Rate (mm/rev)	Depth of Cut (mm)
**Level 1**	30	0.1	0.2
**Level 2**	90	0.3	0.6

## Data Availability

The data presented in this study are available on request from the corresponding author.
